# An ecological model of barriers to accessing care for pregnancy resulting from sexual violence: a rapid review

**DOI:** 10.1186/s12978-025-02189-6

**Published:** 2025-11-12

**Authors:** Paige D. Gilliland, Stephanie D. Ha, Jennifer E. Phipps, Leigh Ann Simmons

**Affiliations:** 1 Department of Human Ecology, University of California, Davis, 1 Shields Ave, , Davis, CA 95616 United States of America; 2 Department of Epidemiology, University of California, Los Angeles, 650 Charles E Young Dr S, Los Angeles, CA 90095 United States of America; 3 Betty Irene Moore School of Nursing, University of California, Davis, 2570 48th St, Sacramento, CA 95817 United States of America

**Keywords:** Sexual violence, Sexual assault, Rape, Pregnancy, Barriers to care

## Abstract

**Background:**

It is estimated that 30% of women globally have experienced sexual violence (SV) and many of these cases result in sexual violence related pregnancies (SVRP). Social and structural inequities may impact access to reproductive healthcare for those who become pregnant as a result of SV.

**Methods:**

Using Bronfenbrenner’s Bioecological Systems Theory as an organizing framework, we conducted a rapid review to identify barriers to accessing reproductive healthcare after SV. We reviewed studies published between 2010 and 2024, synthesizing results from PubMed, PsychInfo, Cumulative Index to Nursing and Allied Health Literature (CINAHL), Educational Resources Information Center (ERIC), Scopus, Global Health, and Embase. A total of 15 studies met the following inclusion criteria: (a) published in a peer-reviewed journal between January 2010 and February 2024, (b) written in English, and (c) evaluated barriers to reproductive healthcare services and resources for people who become pregnant as a result of SV. Study populations were located both within and outside the US.

**Results:**

The primary barriers identified included: lack of access to care, lack of provider training in trauma-informed care, fear of stigmatization, victim blaming, internalized shame, and retaliation.

**Conclusions:**

This review can be used to understand specific barriers to reproductive healthcare for those who have experienced SV and the level or levels at which they are functioning. These findings can be used to tailor reproductive health policy, training, and community-based interventions aimed at mitigating and/or eliminating these barriers.

**Supplementary Information:**

The online version contains supplementary material available at 10.1186/s12978-025-02189-6.

## Background

Sexual violence (SV), including sexual assault, rape, intimate partner SV, and sexual abuse, is a highly prevalent and concerning public health issue globally with 1 in 3 women having experienced some form of SV [1]. Of the cases that result in pregnancy, terminating the pregnancy (i.e., abortion) is more common than continuing the pregnancy [2]. Reproductive rights, including abortion care, are not well protected in the US or globally [3, 4] The overturning of Roe v. Wade has set back reproductive rights, emboldening anti-abortion movements everywhere and strengthening pro-choice advocates by providing them a platform [4]. With new debates and legislation being passed on reproductive healthcare access, it is critical to understand barriers for those in vulnerable or marginalized populations, especially those who have experienced SV [3]. Research on barriers to reproductive healthcare for survivors of SV is severely lacking, leaving current reproductive healthcare policies unbacked by evidence.

The purpose of this paper is to elucidate the barriers that people who become pregnant due to SV face when accessing services and resources. To achieve this goal, we utilized Bronfenbrenner’s Bioecological model [5], which examines how our environment, organized in a series of systems, influences health decision making. By elucidating barriers to reproductive healthcare across levels from the macrosystem (e.g., abortion law) to the individual (e.g., decision making), we can enlist a call to action for research and policy.

## Methods

We conducted a rapid review of the literature examining barriers to care for people who become pregnant due to SV. We chose a rapid review format, because there is minimal existing research in this area, and the quickly shifting political landscape in the past few years has negatively affected reproductive healthcare in the US and beyond [3, 4, 6] Rapid reviews are a rigorous but restricted way to assess empirical evidence related to policy, intervention, or practice using a large body of literature [7]. While they do not currently have a standardized reporting method, we followed a protocol based on guidelines outlined by the World Health Organization (WHO) [7] and the Preferred Reporting Items for Systematic Reviews and Meta-Analyses (PRISMA) checklist [8]. The aims of this rapid review were to: (1) elucidate the barriers pregnant survivors of SV face when accessing reproductive healthcare services and resources; (2) categorize each barrier within the Bioecological Model; and (3) provide recommendations for policy, intervention, and future research.

### Search strategy

A search was conducted using the following databases: PubMed, PsycInfo, Cumulated Index to Nursing and Allied Health Literature (CINAHL), Educational Resources Information Center (ERIC), Scopus, Global Health, and Embase. We used key words for “pregnancy” and “sexual violence”. The search was limited to articles published in English between 2010 and 2024. Results (*n* = 544) were then exported to Covidence software (Veritas Health Innovation, Melbourne, Australia. Available at www.covidence.org), and 243 duplicates were removed. Detailed search strategies are available in Supplemental Table 1. This search was limited to the years 2010–2024 to pull research after the Affordable Care Act, which provided mandated coverage for contraceptive methods, preventative healthcare for women, and improved reproductive healthcare access.

### Study screening

Two reviewers (SH and PG) independently performed a title, abstract, and full-text review based on the established inclusion criteria. Articles were only included if they satisfied all four criteria (Supplemental Table 2): (1) peer-reviewed journal article, (2) written in English, (3) published between January 2010 and February 2024, and (4) evaluated barriers to services and resources for people who became pregnant as a result of experiencing SV. Any discrepancies between reviewers were discussed and resolved.

### Data extraction and analysis

Two reviewers (SH and PG) independently extracted the following information from the included studies: study design, age, demographics, population sample, race/ethnicity, country of study, type of SV, bioecological level, barriers mentioned to accessing resources and services for those who became pregnant because of SV, and any descriptions of providers’ experiences when administering healthcare services to this population. According to Bronfenbrenner, a person’s ecology is topologically referred to as a “nested arrangement of structures,” which go from one system to the next and influence the individual differently [5]. Discrepancies were discussed and then a final extraction was agreed upon by both reviewers.

## Results

### Included studies

**Fig. 1:**
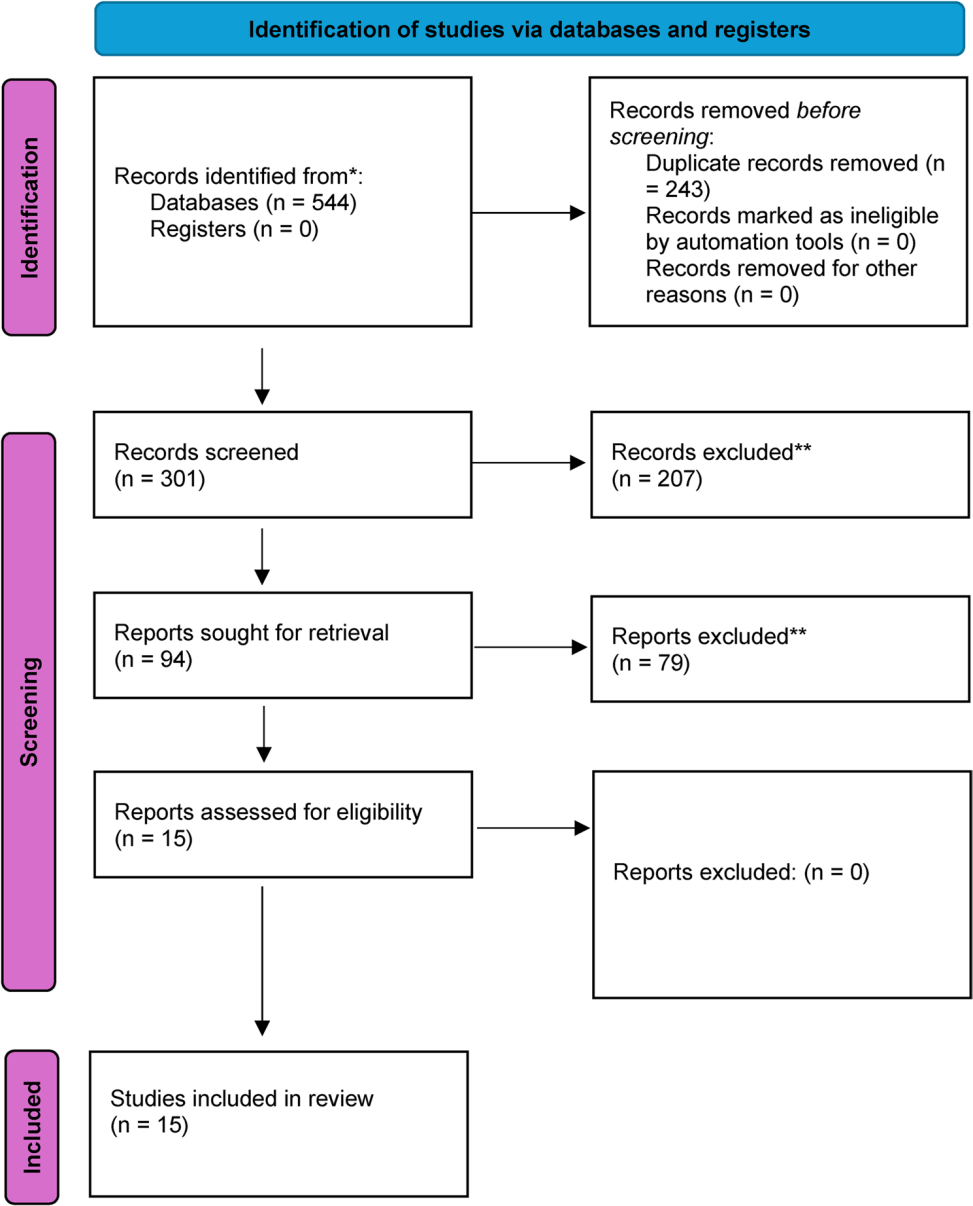
PRISMA Inclusion and Exclusion of Studies. Note. *Records were identified using the search terms and databases in Supplemental Table 1. **Records were excluded based on the list of exclusion criteria in Supplemental Table 2.

In total, 94 studies were evaluated for inclusion; 79 studies were excluded, and 15 studies were included in the final review and synthesis (Supplemental Table 2). The main reason for exclusion was that studies did not focus on the specific population of people who become pregnant because of SV, but instead, examined pregnant populations broadly. The PRISMA flow diagram for the literature search is shown in Fig. 1 and the PRISMA-S checklist is included in supplemental materials.

### Data extraction and quality assessment

Of the included studies, 46% were qualitative studies, and 40% focused their research on a country in Africa (e.g., Uganda). Only two studies focused on the United States and the majority (67%) of the included studies specifically examined barriers to accessing abortion care. Studies reported were conducted in the Democratic Republic of the Congo (DRC) (*n* = 2), Uganda (*n* = 2), United States (*n* = 2), Cameroon (*n* = 1), Chile (*n* = 1), Ethiopia (*n* = 1), and India (*n* = 1). Four articles searched for literature worldwide, and one specifically searched for literature in low/middle-income countries. Contexts included: human trafficking, abortion care, conflict related SV, survival sex, homelessness, and alcohol-involved rape. The quality of each study was assessed using the Joanna Briggs Institute Critical Appraisal Tool for Systematic Reviews and Research Synthesis (JBI) [9] or the Mixed Methods Appraisal Tool (MMAT) for qualitative and quantitative research studies [10]. Quality assessments were completed independently by two reviewers (PG and SH) and a percentage was calculated based on the assessment of each article. The quality percentage from the JBI was (91%) and the quality percentage calculated using the MMAT was (85.7%). See full bias analysis results in Supplemental Tables 3 and 4. See Tables 1 and 2 for a summary of all included articles and findings.

**Table 1:** Summary of included studies

Citation	Title	Objective	Methods	Location	Outcomes
Espinoza et al., 2020 [19]	Abortion knowledge, attitudes and experiences among adolescent girls: A review of the literature	Examine abortion experiences among girls aged 10–14	Systematic Review	LMIC	For girls 10–14, knowledge is lacking on reproductive healthcare access and there is significant stigma/shame
Munro et al., 2021 [22]	Access experiences and attitudes toward abortion among youth experiencing homelessness in the United States: A systematic review	Understand access and attitudes towards abortion among homeless youth in the United States	Systematic Review	United States	Homeless youth experiences substantial barriers to reproductive healthcare in the United States.
Rubini et al., 2023 [16]	Negative consequences of conflict-related sexual violence on survivors: A systematic review of qualitative evidence	Examine negative consequences of conflict-related sexual violence on victims’ health	Systematic Review	International	Many negative impacts to health and barriers to care were identified
Nightingale et al., 2020 [15]	Experiences of pregnancy and maternity care for women exposed to human trafficking and sexual exploitation: a systematic review and qualitative evidence synthesis	To identify and synthesize the experiences of pregnancy and maternity care for those exposed to human trafficking and sexual exploitation.	Systematic Review	Global	Women exposed to human trafficking and sexual exploitation experience many barriers to care and negative impacts to their physical, mental, and sexual health as a result of illness or abuse.
Burtscher et al., 2020 [11]	“Better dead than being mocked”: an anthropological study on perceptions and attitudes towards unwanted pregnancy and abortion in the Democratic Republic of Congo.	To identify barriers to care and understand the health-seeking behaviors of women and girls in the Democratic Republic of Congo who experience unwanted pregnancies.	Qualitative Study	Democratic Republic of the Congo	Perceptions and attitudes towards unwanted pregnancy and abortion varied across regions, but barriers to care were consistently present.
Olson & Kamurari, 2017 [12]	Barriers to safe abortion access: uterine rupture as complication of unsafe abortion in a Ugandan girl.	To identify barriers to safe abortion access for a girl in a rural Ugandan healthcare facility.	Case Report	Uganda	Multiple barriers exist and compound to restrict access to safe abortion care.
Perry et al., 2016 [2]	Practices Regarding Rape-related Pregnancy in U.S. Abortion Care Settings	To explore current practices in U.S. abortion care settings regarding sexual violence screening and clinical responses to the disclosure of rape-related pregnancy.	Quantitative Study	United States	Current practices are variable across care settings and barriers exist in the screening for and disclosures of rape-related pregnancy.
Woldetsadik et al., 2022 [17]	The enduring consequences of conflict-related sexual violence: a qualitative study of women survivors in northern Uganda.	To understand women’s perceptions of and experiences with conflict-related sexual violence in Northern Uganda.	Qualitative Study	Uganda	Women are impacted physically and psychologically as a result of conflict-related sexual violence, and many experience barriers to seeking care.
Montero et al., 2023 [14]	Main barriers to services linked to voluntary pregnancy termination on three grounds in Chile.	To identify the barriers hindering access to services provided by decriminalizing voluntary termination of pregnancy in Chile.	Qualitative Study	Chile	The main barriers identified were barriers related to accessing resources, barriers after women have entered the healthcare system, and lack of effectiveness of resources.
Davis et al., 2024 [21]	Alcohol-involved rape: Limitations of the “rape exception” for abortion access.	To examine research on alcohol-involved rape and identify barriers to abortion service access through rape exceptions.	Systematic Review	United States	Victim alcohol intoxication may prevent the use of rape exceptions for abortion access.
Greer et al., 2023 [24]	Barriers to reporting and lack of equitable support: Abortion access for adults with autism experiencing rape-related pregnancy post-roe	To examine the barriers and lack of equitable support that individuals with autism experience regarding abortion access.	Systematic Review	United States	Individuals with autism experience many barriers to abortion access and support after experiencing rape-related pregnancy.
O’Connell et al., 2022 [13]	Signs of a turning tide in social norms and attitudes toward abortion in Ethiopia: Findings from a qualitative study in four regions.	To explore the social barriers that women experience seeking abortion care in Ethiopia	Qualitative Study	Ethiopia	Many barriers, including stigmatization, exist that prevent women in Ethiopia from accessing abortion care.
Scott et al., 2018 [23]	A qualitative analysis of decision-making among women with sexual violence-related pregnancies in conflict-affected eastern Democratic Republic of the Congo.	To understand decision-making among women who have sexual-violence related pregnancies in the Democratic Republic of the Congo.	Qualitative Study	Democratic Republic of the Congo	There are many influences on decision-making, which can be grouped into the identities of the fetus/future child, social reactions, and the power of religious and moral beliefs.
Schuster et al., 2010 [18]	Women’s experiences of the abortion law in Cameroon: “what really matters”.	To examine women’s experiences of Cameroon’s abortion law	Qualitative Study	Cameroon	Women’s experiences are described, summarizing the harsh circumstances in which they become pregnant and the difficulties seeking abortion care.
Subramaniyan et al., 2017 [20]	Barriers and Challenges in Seeking Psychiatric Intervention in a General Hospital, by the Collaborative Child Response Unit, (A Multidisciplinary Team Approach to Handling Child Abuse) A Qualitative Analysis.	To understand how different sectors of the hospital collaborate to provide care for survivors of child abuse and explore barriers to integration of psychiatric care.	Qualitative Study	India	Many barriers to psychiatric care were identified, including stigma and victim blaming for terminating the pregnancy.

**Table 2:** Critical findings organized with bronfenbrenner’s ecological model

Macrosystem	Fear of law enforcement, lack of access to safe services, and the mandatory reporting of rape in cases with minors.
**Exosystem**	Lack of ID in cases with human trafficking survivors, lack of interpreters, lack of protocol for rape-related pregnancies, cost barriers, low-quality emergency services, and lack of follow up services.
**Mesosystem**	Providers working with perpetrators, lack of non-shameful or trusting experiences with providers, fear of negative reputation, lack of empathy from providers, providers refusing to provide care due to religious, personal, or discriminatory beliefs, and harmful stereotypes around alcohol related rape.
**Microsystem**	Fear of rejection from family, peers, and partners, lack of “physical” evidence after SV.
**Individual**	Compounding shame around abortion and sexual violence, fearing retaliation from perpetrator, and self-blame.

### Macrosystem level

The macrosystem refers to the social mores, economic systems (e.g., nationwide economic policies and systems), culture, and politics of where the individual who experienced SV resides. Burtscher and colleagues [11] interviewed healthcare providers, community leaders, religious leaders, local authorities, women who had experienced SV or abortion, and the public in the DRC. Unwanted pregnancy and abortion are highly sensitive topics, and the stigma surrounding them creates a culture where women keep their pregnancies secret and rely on healthcare that is suboptimal [11–13]. Montero and colleagues [14] found that in Chile, one major barrier to accessing care is that it is mandatory to report rape to the authorities in cases with minors and to receive permission for termination of pregnancy. Another barrier within the macrosystem revolved around criminalization and discrimination. In a study conducted in the U.S. with sex trafficking survivors, researchers reported that many survivors refrained from sharing crucial information because they were concerned that the provider would report the assault to the authorities, which could potentially result in their arrest due to the criminalization of sex work [15]. 

### Exosystem level

The exosystem refers to the local governments, economic systems (e.g., regional economic policies and systems), and other authoritative entities within a macrosystem that encompasses the individual who experienced SV. Nightingale and colleagues [15] interviewed victims of human trafficking who had become pregnant because of SV. They reported that many healthcare providers are not aware of the rights and resources to which human trafficking survivors are entitled, and these survivors are sometimes turned away as a result [15]. For example, some survivors stated that they were turned away for not providing appropriate proof of identification. For people who have limited English proficiency, a lack of available interpreters impacted their willingness and ability to disclose sensitive information [15]. One study evaluated U.S. abortion policies by surveying abortion providers to better understand how they screen for rape-related pregnancies, provide support, and if they have had encounters with law enforcement [2]. Over half of the respondents reported issues identifying those who had experienced rape-related pregnancy, and only 19.7% reported having specific protocols in place for handling rape-related pregnancies [2]. One systematic review found that lack of follow-up care, poor clinician-to-patient ratios, low-quality emergency services, and lack of healthcare staff training were all significant barriers to accessing care post-SV [16]. Woldetsadik et al. [17] interviewed female survivors from post-conflict regions in Northern Uganda about their experience with conflict-related sexual violence. All participants in this study had been abducted between 1989 and 2005 for anywhere between 2 weeks and 9 years with an average length of abduction being 4.7 years [17]. Most stated that they did not continue seeking healthcare services after the initial appointment directly after the SV. This was largely due to fear and anxiety about sharing their experience, not knowing where to go, cost barriers, and a fear of retaliation or using their SV experience against them [16, 17].

### Mesosystem

One of the more complex systems within Bronfenbrenner’s Bioecological Model is the mesosystem. This system is the interaction between the microsystem and exosystem, and the extent to which healthcare providers, local agencies/policies, media, and extended families influence the individual who experienced SV’s immediate relationships. The religiosity of an area influences community-level feelings and beliefs about abortion, which directly impact the provision of services, as is the case for Catholic hospitals that do not provide abortion care [11, 12] Research has shown that some healthcare providers have refused to provide abortion services for rape-related abortion due to personal or political beliefs [12]. Further, some survivors reported experiencing refusal of care due to homophobic, xenophobic, and racist healthcare staff [16]. Many healthcare staff lacked empathy and engaged in victim blaming [16]. Additionally, some people reported that a lack of “physical” evidence was the reason they were not believed [16]. 

### Microsystem

The microsystem refers to the closest inner circle of the individual who experienced SV such as their families and friends. Many young women who become pregnant because of nonconsensual sex state that they cannot tell their friends or family out of fear of rejection [11, 16, 18, 19]. In Northern Uganda, around two-thirds of the women said that they had been rejected by family members due to their abduction and abuse [17]. Many of the husbands or close family were emotionally abusive or blamed them for their experience with SV [11, 16, 20]. All the women shared that their relationships with their current partners were affected by their experience with SV [17]. One woman stated that her partner sometimes uses her history of being raped against her in arguments to belittle her [17]. 

### Individual system

The individual system encompasses sex, gender, age, biology, and the inner psychological experiences individuals who experienced SV face because of the other four systems. Primary findings in this system include fear of repercussions from perpetrators (e.g., traffickers and authorities) and the compounding internalized shame associated with stigmas surrounding abortion, sex work, and alcohol-involved rape [13, 15, 16, 21–23]. Some survivors report their belief that experiencing SV is a “normal” part of a woman’s life, inhibiting their desire to access care [16]. In Ethiopia, some individuals reported they would “compound” the shame surrounding rape by seeking out abortion services. One respondent stated they would support abortion in cases of rape-related pregnancy only if there was “proof” of the rape [13]. The same participant also stated this “proof” needed to demonstrate that this survivor was not “on drugs,” further demonstrating the significant blame and shame surrounding SV and rape [13]. In another study conducted in the DRC, many of the women stated they feared social rejection due to the stigmatization of abortion and SV [23].

Youth experiencing homelessness who have experienced SV highlighted the importance of having experiences with healthcare providers that are not shameful [22]. One person mentioned they have internalized stigmas surrounding “survival sex” and look down on their peers for engaging in it even though it is nonconsensual [22]. In the same study, many participants reported performing self-induced abortions due to the shame and stigma surrounding abortion and rape, even in states with more supportive abortion policies [22]. One survivor of human trafficking explained that if they were to become pregnant, or even miss their period, they would fear that they would be beaten by their perpetrators (e.g., captors or traffickers) [15]. Beatings could be incurred for growing a large belly, working less, losing income, and not having enough money to cover an abortion [15]. In many cases, women who have survived or are currently victims of human trafficking are aware that they are being judged as sex workers [15]. In situations with alcohol-involved rape, women are particularly likely to engage in self-blame due to the stigmatization of intoxicated rape and lack of consistent legal definitions around consent [21]. 

Autistic individuals who have experienced SV often have increased difficulty recognizing that what they experienced was SV [24]. Many autistic women reported fearing physical examinations or being touched as this is often extremely overstimulating for neurodivergent people [24]. The individual system encompasses patient/participant lived experience and often reflects barriers experienced at all other levels of the bioecological model. Addressing barriers faced at all other levels can greatly reduce the barriers faced at the individual level (i.e., shame, fear, and stigma).

## Discussion and future directions

This rapid review employed Bronfenbrenner’s Bioecological Model to fill a significant gap in identifying barriers to reproductive health care for people who become pregnant due to SV. Despite the impact of the US policies and trends on global health, only two studies were conducted in the US, and both specifically focused on experiences of accessing abortion care [2, 22]. Further, the research is disjointed. Very few studies examining SV also investigated sexual violence related pregnancy (SVRP), and studies examining barriers to perinatal care rarely included SVRP. Given that the US often influences other nations’ reproductive healthcare policies and programs, it is important to prioritize and conduct research on reproductive healthcare access for all populations. By conducting theory-based research on reproductive healthcare access, we can continue to influence policy impacting women and people in and outside of the US. For example, by conducting research grounded in health equity and reproductive justice, we may be able to develop specialized training, interventions, and policies that increase perinatal healthcare access to those who have experienced SV.

This study serves as a guide to understanding specific barriers that exist for those who have become pregnant because of SV and the level or levels at which they are functioning. These findings can be used to promote policies and community-based interventions aimed at mitigating and/or eliminating these barriers. Some potential policy, research, and practice implications include increased training of providers, law enforcement, and others who work with survivors of SV, sex education that focuses on consent, anti-sexual violence campaigns, family-based interventions, and more research on barriers to reproductive healthcare access. By increasing the training of police officers, healthcare staff, and other practitioners in SV-related care [21], we may be able to reduce the incidence of re-traumatization and decrease maternal morbidity and mortality in cases of SVRP. There are international trainings available online from the International Trauma Training Institute [25] and International Association of Forensic Nurses [26]. We also can increase comprehensive sexual health education to destigmatize abortion and sexual health, overall. In the United States, United Kingdom, and Australia, policymakers have enacted requirements for schools and universities to teach consent [27]. Some programs have started to incorporate the risk of intoxication and more awareness about the role of intoxication in sexual violence [28]. Additionally, there is a call for consent to be taught alongside other school topics and in school-wide assemblies to enact more cultural change [28]. 

### Strengths and limitations

This review has several strengths and limitations. Our findings were limited to seven databases, which could have excluded relevant studies. In addition, there was a gap in the literature examining the intersections of pregnancy and SV. Out of all the research cited, only seven countries were represented, limiting the generalizability of the findings, as different countries have different policies, practices, laws, and cultures that can affect the barriers that people experience. This article has the capacity to influence how researchers and policymakers think about sexual health-related policies and the experiences of those who become pregnant because of SV.

## Conclusions

To our knowledge, this is the first rapid review to examine the barriers to reproductive healthcare for those who have experienced sexual violence (SV) using Bronfenbrenner’s Bioecological model. The barriers to access defined in this review reflect macro-, exo-, meso-, and microsystemic, as well as individual level factors, influencing people’s access to reproductive healthcare after SV. By organizing these barriers using Bronfenbrenner’s guiding framework, we can better examine gaps in research, policy, and intervention at multiple levels and understand at what level these barriers are influencing people. Importantly, this approach can support multi-level interventions that may be more impactful for survivors of SV. 

## Supplementary Information

Supplementary Material 1

Supplementary Material 2

## Data Availability

Not applicable.
