# Correction: An ecological model of barriers to accessing care for pregnancy resulting from sexual violence: a rapid review

**DOI:** 10.1186/s12978-025-02237-1

**Published:** 2025-12-18

**Authors:** Paige D. Gilliland, Stephanie D. Ha, Jennifer E. Phipps, Leigh Ann Simmons

**Affiliations:** 1 Department of Human Ecology, University of California, Davis, 1 Shields Ave, Davis, CA 95616 United States of America; 2 Department of Epidemiology, University of California, Los Angeles, 650 Charles E Young Dr S, Los Angeles, CA 90095 United States of America; 3 Betty Irene Moore School of Nursing, University of California, Davis, St, Sacramento, 2570 48th, 95817 CA United States of America

**Correction: Gilliland et al. Reproductive Health 22, 225 (2025)**

**https://doi.org/10.1186/s12978-025-02189-6**

In this article [1], the author contribution statement was incorrect in the originally published article. The correct author contribution should have appeared as shown below:

Authors’ contributions.

P.G and S.H. wrote the main manuscript text, prepared the figure, tables, and supplemental information. J.P. and L.A.S. contributed to study conceptualization, design, drafting, editing, and preparing the manuscript for publication. All authors reviewed the full manuscript and materials prior to submission.

The original article has been corrected.
