# Supplementary material for: An ecological model of barriers to accessing care for pregnancy resulting from sexual violence: a rapid review
Source: Reprod Health. 2025 Nov 12;22:225. doi: 10.1186/s12978-025-02189-6 (PMC12607059; doi:10.1186/s12978-025-02189-6)
Supplement: Supplementary file 1 — Supplementary Material 1 [file 12978_2025_2189_MOESM1_ESM.docx]

**Supplemental Table 1. Search Strategy**

**Methods:**

A search was executed by an expert searcher/health librarian on the following databases:  PubMed, PsychInfo, CINAHL, ERIC, Scopus, Global Health, and Embase.

**Date of last search:** February 21, 2024

| Database | Subject index terms | Results |
| --- | --- | --- |
| PubMed | ((treatment barriers[Title/Abstract] OR barriers[Title/Abstract] OR healthcare access[Title/Abstract]) AND (sexual abuse[Title/Abstract] OR rape[Title/Abstract] OR sexual violence[Title/Abstract] OR sexual assault[Title/Abstract] OR intimate partner sexual violence[Title/Abstract]) AND (pregnancy[Title/Abstract] OR perinatal period[Title/Abstract] OR pregnant[Title/Abstract] OR pre-natal[Title/Abstract] OR prenatal[Title/Abstract]) | 14 |
| Psycinfo | (noft("Intimate Partner Sexual Violence" OR "sexual abuse" OR "sexual violence" OR "sexual assault" OR "rape" OR "intimate partner sexual violence") OR (MJMAINSUBJECT.EXACT("Sexual Assault") OR MJMAINSUBJECT.EXACT(“Rape”) OR MJMAINSUBJECT.EXACT("Intimate Partner Sexual Violence") OR MJMAINSUBJECT.EXACT("Sexual Violence") OR MJMAINSUBJECT.EXACT("Sexual Abuse"))) AND (noft( "Perinatal Period" OR "pregnanc*" OR "pre-natal" OR "prenatal") OR (MJMAINSUBJECT.EXACT("Pregnancy") OR MAINSUBJECT.EXACT("Prenatal Care") OR MAINSUBJECT.EXACT("Reproductive Health") OR MAINSUBJECT.EXACT("Perinatal Period"))) AND (noft("treatment barriers" OR "barriers") OR (MJMAINSUBJECT.EXACT("Health Care Access") OR MJMAINSUBJECT.EXACT("Treatment Barriers") OR MJMAINSUBJECT.EXACT("Health Care Delivery") OR MJMAINSUBJECT.EXACT(“Healthcare Access”))) | 12 |
| Cinahl | ( ( (MM "Communication Barriers") OR (MM "Health Services Accessibility") OR (MM "Attitude to Medical Treatment") OR (MM "Attitude of Health Personnel") ) OR TI ( "treatment barriers" OR "barriers" ) OR AB ( "treatment barriers" OR "barriers" ) OR TI (“healthcare access”) OR AB (“healthcare access”)) AND ( ( TI ( "pregnancy" or "pregnant" or "prenatal" or "perinatal period" or "pre-natal" ) ) OR ( AB ( "pregnancy" or "pregnant" or "prenatal" or "perinatal period" or "pre-natal" ) ) OR ( MM "Pregnancy in Adolescence") OR (MM "Pregnancy, Unplanned") OR (MM "Pregnancy Outcomes") OR (MM "Pregnancy Discomforts") OR (MM "Attitude to Pregnancy") OR (MM "Perinatal Period") OR (MM "Pregnancy, Unwanted") OR (MM "Pregnancy Care (Saba CCC)") OR (MM "Pregnancy") OR (MM "Risk Control: Unintended Pregnancy (Iowa NOC)") OR (MM "Expectant Mothers") OR (MM "Family Planning") ) ) AND ( (MM "Intimate Sexual Partner Violence") OR (MM "Sexual Assault") OR (MM "Exposure to Sexual Violence") OR (MM "Sexual Partners") OR (MM "Sexual Abuse") OR (MM "Sexual Assault Examination") OR (MM "Child Abuse, Sexual") OR (TI ( "Intimate Partner Sexual Violence" OR "sexual abuse" OR "sexual violence" OR "sexual assault" OR "rape" OR "sexual violence" OR "intimate partner sexual violence" OR "partner abuse" )) OR (AB ( "Intimate Partner Sexual Violence" OR "rape" OR "sexual violence" OR "sexual assault" OR "sexual abuse" OR "intimate partner sexual violence")) ) | **5** |
| Scopus* | ( TITLE-ABS-KEY ( "treatment barriers"  OR  "barriers" OR “healthcare access” )  AND  TITLE-ABS-KEY ( "pregnancy"  OR  "perinatal period"  OR  "pregnan*"  OR  "prenatal"  OR  "pre-natal" )  AND  TITLE-ABS-KEY ( "Intimate Partner Sexual Violence"  OR  "sexual abuse"  OR  "sexual violence"  OR  "sexual assault"  OR  "rape" OR  "intimate partner sexual violence”) )    “Intimate Partner Sexual Violence” OR “sexual abuse” OR “sexual violence” OR “sexual assault” OR “rape” OR “intimate partner sexual violence” | 33 |
| ERIC | ((MJMAINSUBJECT.EXACT("Sexual Abuse") OR MAINSUBJECT.EXACT("Rape")) OR noft("Intimate Partner Sexual Violence" OR "sexual abuse" OR "sexual violence" OR "sexual assault" OR "intimate partner sexual violence")) AND (noft("pregnancy" or "pregnant" or "prenatal" or "perinatal period" or "pre-natal") OR (MJMAINSUBJECT.EXACT("Pregnancy") OR MJMAINSUBJECT.EXACT("Pregnant Students"))) AND ((MJMAINSUBJECT.EXACT("Access to Education") OR MJMAINSUBJECT.EXACT("Access to Information") OR MJMAINSUBJECT.EXACT("Barriers") OR MJMAINSUBJECT.EXACT("Access to Health Care")) OR noft("treatment barriers" OR "barriers")) | 0 |
| Global Health | (title:("Intimate Partner Sexual Violence" OR "sexual abuse" OR "sexual violence" OR "sexual assault" OR "rape" OR "intimate partner sexual violence") OR ab:("Intimate Partner Sexual Violence" OR "sexual abuse" OR "sexual violence" OR "sexual assault" OR "rape" OR "intimate partner sexual violence") OR subject:("Intimate Partner Sexual Violence" OR "sexual abuse" OR "sexual violence" OR "sexual assault" OR "rape" OR "intimate partner sexual violence")) AND (title:("pregnancy" OR "pregnant adolescent" OR "pregnant women" OR "prenatal care" OR "prenatal period" OR "perinatal period" OR "pregnant" or "prenatal" OR "pre-natal") OR ab:("pregnancy" OR "pregnant adolescent" OR "pregnant women" OR "prenatal care" OR "prenatal period" OR "perinatal period" OR "pregnant" or "prenatal" OR "pre-natal") OR subject:("pregnancy" OR "pregnant adolescent" OR "pregnant women" OR "prenatal care" OR "prenatal period" OR "perinatal period" OR "pregnant" or "prenatal" OR "pre-natal")) AND (title:("barriers" OR "social barriers" OR "treatment barriers" OR "barriers") OR ab:("barriers" OR "social barriers" OR "treatment barriers" OR "barriers") OR subject:("barriers" OR "social barriers" OR "treatment barriers" OR "barriers")) | 0 |
| Embase | ('treatment barriers':ti OR 'barriers':ti OR 'treatment barriers':ab OR 'barriers':ab OR 'treatment barriers' OR 'barriers'/mj OR ‘healthcare access’:ab OR ‘healthcare access’:ti) AND ("pregnancy":ti or "pregnant":ti or "prenatal":ti or "perinatal period":ti or "pre-natal":ti OR "pregnancy":ab or "pregnant":ab or "prenatal":ab or "perinatal period":ab or "pre-natal":ab OR "pregnancy"/mj or "pregnant"/mj or "prenatal"/mj or "perinatal period"/mj or "pre-natal"/mj) AND ("Intimate Partner Sexual Violence":ti OR "sexual abuse":ti OR "sexual violence":ti OR "sexual assault":ti OR "rape":ti OR "intimate partner sexual violence":ti OR "Intimate Partner Sexual Violence":ab OR "sexual abuse":ab OR "sexual violence":ab OR "sexual assault":ab OR "rape":ab OR "intimate partner sexual violence":ab OR "Intimate Partner Sexual Violence"/mj OR "sexual abuse"/mj OR "sexual violence"/mj OR "sexual assault"/mj OR "rape"/mj OR "intimate partner sexual violence"/mj) | 8 |

**Table 2. Inclusion and exclusion criteria**

|  | **Inclusion criteria** | **Exclusion criteria** |
| --- | --- | --- |
| **Study design** | Peer reviewed primary empirical studies (qualitative, quantitative, or mixed methods), theoretical studies; reviews of empirical studies; implementation studies | Commentaries, editorials, review protocols |
| **Population** | Those who became pregnant because of SV | No pregnancy because of SV |
| **Setting** | Perinatal care | Non-perinatal care |
| **Time** | Limited to studies published after January 2010 | Articles published prior to February 2024 |

**Table 3**. Quality assessment for included reviews using the JBI Critical Appraisal Checklist

|  | Espinoza et al., 2020 | Munro et al., 2021 | Nightingale et al., 2020 | Fornari et al., 2022 | Rubini et al., 2023 | % |
| --- | --- | --- | --- | --- | --- | --- |
| Is the review question clearly and explicitly stated? | Yes | Yes | Yes | Yes | Yes | 100% |
| Were the inclusion criteria appropriate for the review question? | Yes | Yes | Yes | Yes | Yes | 100% |
| Was the search strategy appropriate? | Yes | Yes | Yes | Yes | Yes | 100% |
| Were the sources used to search for studies adequate? | Yes | Yes | Yes | Yes | Yes | 100% |
| Were the criteria for appraising studies appropriate? | Yes | Yes | Yes | Unclear | Yes | 80% |
| Was critical appraisal conducted by two or more reviewers independently? | Yes | Yes | Yes | No | Yes | 80% |
| Were there methods to minimize errors in data extraction? | Yes | Yes | Yes | No | Yes | 80% |
| Were the methods used to combine studies appropriate? | Yes | Yes | Yes | Yes | Yes | 100% |
| Was the likelihood of publication bias assessed? | Yes | Yes | Yes | No | No | 60% |
| Were recommendations for policy and/or practice supported by the reported data? | Yes | Yes | Yes | Yes | Yes | 100% |
| Were the specific directives for new research appropriate? | Yes | Yes | Yes | Yes | Yes | 100% |
| Quality Score | 100% | 100% | 100% | 63.6% | 91% | 91% |

*Note.* The JBI checklist uses a scale of Yes, No, Unclear, or Not Applicable. References are from the main manuscript.

**Table 4.** Quality assessment for included quantitative and qualitative studies using the MMAT checklist

| **Qualitative** | Are there clear research questions? | Do the collected data allow to address the research questions? | Does the qualitative approach answer the research questions? | Are the qualitative methods able to address the research question? | Are the findings adequately derived from the data? | Is the interpretation of results sufficiently substantiated by data? | Is there coherence between qualitative data sources & interpretation? | Quality Score |
| --- | --- | --- | --- | --- | --- | --- | --- | --- |
| Scott et al., 2018 | Yes | Yes | Yes | Yes | Yes | Yes | Yes | 100% |
| Subramaniyan et al., 2017 | Yes | Yes | Yes | Yes | Yes | Yes | Yes | 100% |
| Olson & Kamurari, 2017 | No | No | Yes | Yes | No | No | No | 28.5% |
| Burtscher et al., 2020 | Yes | Yes | Yes | Yes | Yes | Yes | Yes | 100% |
| O'Connell et al., 2022 | Yes | Yes | Yes | Yes | Yes | Yes | Yes | 100% |
| Woldetsadik et al., 2022 | Yes | Yes | Yes | Yes | Yes | Yes | Yes | 100% |
| Schuster, 2010 | Yes | Unsure | Yes | No | Yes | No | Yes | 57% |
| Montero et al., 2023 | Yes | Yes | Yes | Yes | Yes | Yes | Yes | 100% |
| Greer et al., 2023 | Yes | Unsure | Yes | Unsure | Yes | Yes | Yes | 71.4% |
| **Quantitative** | Are there clear research questuins? | Do the collected data address the research questions? | Is the sampling relevant to the research question? | Is the sample representative of the target population? | Measurements are appropriate? | Is the risk of nonresponse bias low? | Is the statistical analysis appropriate to answer the research question? | Quality Score |
| Perry et al., 2016 | Yes | Yes | Yes | Yes | Yes | Yes | Yes | 100% |
| % | 90% | 70% | 100% | 80% | 90% | 80% | 90% | 85.7% |

*Note.* The MMAT checklist uses a scale of Yes, No, or Unsure. References are from main manuscript.

|  |
| --- |
|  |
|  |
|  |
|  |
|  |
|  |
|  |
|  |
|  |
|  |
|  |
|  |
